# 3-Amino-1-(4-bromo­phen­yl)-9,10-dihydro­phenanthrene-2,4-dicarbonitrile

**DOI:** 10.1107/S1600536811038517

**Published:** 2011-09-30

**Authors:** Abdullah M. Asiri, Hassan M. Faidallah, Abdulrahman O. Al-Youbi, Seik Weng Ng

**Affiliations:** a Chemistry Department, Faculty of Science, King Abdulaziz University, PO Box 80203 Jeddah, Saudi Arabia; b Center of Excellence for Advanced Materials Research, King Abdulaziz University, PO Box 80203 Jeddah, Saudi Arabia; c Department of Chemistry, University of Malaya, 50603 Kuala Lumpur, Malaysia

## Abstract

In the title compound, C_22_H_14_BrN_3_, the fused-ring system is buckled owing to the ethyl­ene linkage in the central ring; the two flanking aromatic rings are twisted by 25.9 (1) ° with respect to each other. The phenyl ring is twisted by 77.0 (1)° relative to the amino- and cyano-bearing aromatic ring. In the crystal, adjacent mol­ecules are linked by two N–H⋯N hydrogen bonds, generating a zigzag chain along [101].

## Related literature

For two related compounds, see: Asiri *et al.* (2011*a* ▶,*b* ▶).
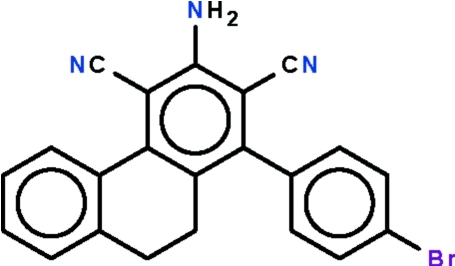


## Experimental

### 

#### Crystal data

C_22_H_14_BrN_3_
*M* *_r_* = 400.27
Monoclinic, 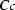

*a* = 13.7683 (5) Å
*b* = 16.2557 (3) Å
*c* = 9.7945 (4) Å
β = 127.546 (6)°
*V* = 1738.07 (17) Å^3^
*Z* = 4
Cu *K*α radiation
μ = 3.29 mm^−1^
*T* = 100 K
0.20 × 0.20 × 0.20 mm

#### Data collection

Agilent SuperNova Dual diffractometer with Atlas detector
Absorption correction: multi-scan (*CrysAlis PRO*; Agilent, 2010 ▶) *T*_min_ = 0.559, *T*_max_ = 0.559
2976 measured reflections
2195 independent reflections
2187 reflections with *I* > 2σ(*I*)
*R*_int_ = 0.012

#### Refinement

*R*[*F* ^2^ > 2σ(*F* ^2^)] = 0.021
*wR*(*F* ^2^) = 0.056
*S* = 1.08
2195 reflections
243 parameters
2 restraints
H atoms treated by a mixture of independent and constrained refinement
Δρ_max_ = 0.22 e Å^−3^
Δρ_min_ = −0.61 e Å^−3^
Absolute structure: Flack (Flack, 1983 ▶), 482 Friedel pairs
Flack parameter: −0.024 (14)

### 

Data collection: *CrysAlis PRO* (Agilent, 2010 ▶); cell refinement: *CrysAlis PRO*; data reduction: *CrysAlis PRO*; program(s) used to solve structure: *SHELXS97* (Sheldrick, 2008 ▶); program(s) used to refine structure: *SHELXL97* (Sheldrick, 2008 ▶); molecular graphics: *X-SEED* (Barbour, 2001 ▶); software used to prepare material for publication: *publCIF* (Westrip, 2010 ▶).

## Supplementary Material

Crystal structure: contains datablock(s) global, I. DOI: 10.1107/S1600536811038517/bt5646sup1.cif

Structure factors: contains datablock(s) I. DOI: 10.1107/S1600536811038517/bt5646Isup2.hkl

Supplementary material file. DOI: 10.1107/S1600536811038517/bt5646Isup3.cml

Additional supplementary materials:  crystallographic information; 3D view; checkCIF report

## Figures and Tables

**Table 1:** Hydrogen-bond geometry (Å, °)

*D*—H⋯*A*	*D*—H	H⋯*A*	*D*⋯*A*	*D*—H⋯*A*
N2—H1⋯N1^i^	0.93 (3)	2.23 (3)	3.097 (3)	155 (3)
N2—H2⋯N3^ii^	0.88 (4)	2.54 (4)	3.307 (3)	147 (3)

Symmetry codes: (i) 
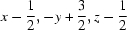
; (ii) 
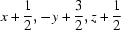
.

**Fig. 1.**
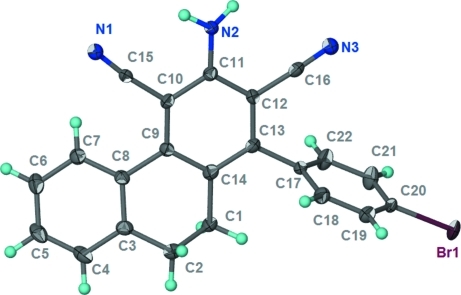
Anisotropic displacement ellipsoid plot (Barbour, 2001) of C22H14N3Br at the 70% probability level; hydrogen atoms are drawn as spheres of arbitrary radius.

## supplementary crystallographic information

### Comment

2-Amino-4-aryl-5,6-dihydrobenzoquinoline-3-carbonitrile is synthesized from the reaction of the α-substituted cinnamonitrile, C_6_H_5_CH═C(CN)_2_, with α-tetralone in a reaction that is catalyzed by ammonium acetate. The synthesis when conducted under microwave irradiation leads to an improved yield. In previous studies, we obtained instead di-carbonitrile substituted dihydrophenanthrenes (3-amino-1-(4-methoxyphenyl)-9,10- dihydrophenanthrene-2,4-dicarbonitrile and 3-amino-1-(2*H*-1,3-benzodioxol-5-yl)- 9,10-dihydrophenanthrene-2,4-dicarbonitrile) with 4-methoxybenzaldehyde and piperonaldehyde in syntheses that differed slightly from the reported ones as we used substituted benzaldehydes, α-tetralone and ethyl cyanoacetate along with a molar excess of ammonium acetate (Asiri *et al.*, 2011*a*; 2011*b*). The use of 4-bromobenzaldehyde furnishes the corresponding 4-bromophenyl analog (Scheme I, Fig. 1). The fused-ring system is buckled owing to the ethylene linkage in the central ring; the two flanking aromatic rings are twisted by 25.9 (1) °. Relative to the amino- and cyano-bearing aromatic ring, the phenyl ring is twisted by 77.0 (1) °. Adjacent molecules are linked by two N–H···N hydrogen bonds to generate a chain along [1 0 1] (Table 1).

### Experimental

4-Bromobenzaldehyde (1.85 g,10 mmol), 1-tetralone (1.46 g, 10 mmol), malononitrile (0.66 g, 10 mmol) and ammonium acetate (6.2 g, 80 mmol) in absolute ethanol (50 ml) were heated for 6 h. The mixture was allowed to cool, and the precipitate was collected, washed with water, dried and then recrystallized from ethanol; m.p. 517–518.

### Refinement

Carbon-bound H-atoms were placed in calculated positions [C–H 0.95 to 0.99 Å, *U*_iso_(H) 1.2–1.5*U*_eq_(C)] and were included in the refinement in the riding model approximation. The amino H atoms were located in a difference Fourier map and were refined freely.

The Flack parameter was refined from 482 Friedel pairs; although the Friedel coverage is low (27%), the Flack parameter was reliably refined owing to the heavy atom.

### Crystal data

**Table:**

C_22_H_14_BrN_3_	*F*(000) = 808
*M**_r_* = 400.27	*D*_x_ = 1.530 Mg m^−^^3^
Monoclinic, *C**c*	Cu *K*α radiation, λ = 1.54184 Å
Hall symbol: C -2yc	Cell parameters from 2539 reflections
*a* = 13.7683 (5) Å	θ = 4.9–74.2°
*b* = 16.2557 (3) Å	µ = 3.29 mm^−^^1^
*c* = 9.7945 (4) Å	*T* = 100 K
β = 127.546 (6)°	Prism, orange
*V* = 1738.07 (17) Å^3^	0.20 × 0.20 × 0.20 mm
*Z* = 4	

### Data collection

**Table:**

Agilent SuperNova Dual diffractometer with Atlas detector	2195 independent reflections
Radiation source: SuperNova (Cu) X-ray Source	2187 reflections with *I* > 2σ(*I*)
Mirror	*R*_int_ = 0.012
Detector resolution: 10.4041 pixels mm^-1^	θ_max_ = 74.4°, θ_min_ = 4.9°
ω scan	*h* = −17→16
Absorption correction: multi-scan (*CrysAlis PRO*; Agilent, 2010)	*k* = −11→20
*T*_min_ = 0.559, *T*_max_ = 0.559	*l* = −11→12
2976 measured reflections	

### Refinement

**Table:**

Refinement on *F*^2^	Secondary atom site location: difference Fourier map
Least-squares matrix: full	Hydrogen site location: inferred from neighbouring sites
*R*[*F*^2^ > 2σ(*F*^2^)] = 0.021	H atoms treated by a mixture of independent and constrained refinement
*wR*(*F*^2^) = 0.056	*w* = 1/[σ^2^(*F*_o_^2^) + (0.0393*P*)^2^ + 0.2403*P*] where *P* = (*F*_o_^2^ + 2*F*_c_^2^)/3
*S* = 1.08	(Δ/σ)_max_ = 0.001
2195 reflections	Δρ_max_ = 0.22 e Å^−^^3^
243 parameters	Δρ_min_ = −0.61 e Å^−^^3^
2 restraints	Absolute structure: Flack (Flack, 1983), 482 Friedel pairs
Primary atom site location: structure-invariant direct methods	Flack parameter: −0.024 (14)

### Fractional atomic coordinates and isotropic or equivalent isotropic displacement parameters (Å^2^)

**Table:**

	*x*	*y*	*z*	*U*_iso_*/*U*_eq_	
Br1	0.00008 (3)	0.303704 (13)	0.00088 (3)	0.02570 (9)	
N1	0.8004 (2)	0.69430 (12)	1.0721 (3)	0.0189 (4)	
N2	0.5320 (2)	0.70911 (13)	0.6833 (3)	0.0173 (4)	
H1	0.460 (3)	0.739 (2)	0.618 (4)	0.018 (7)*	
H2	0.597 (3)	0.738 (2)	0.761 (5)	0.024 (8)*	
N3	0.2436 (2)	0.64780 (13)	0.3201 (3)	0.0245 (5)	
C1	0.5286 (2)	0.36690 (15)	0.8140 (3)	0.0172 (5)	
H1A	0.4753	0.3368	0.7036	0.021*	
H1B	0.4964	0.3581	0.8797	0.021*	
C2	0.6591 (2)	0.33350 (15)	0.9166 (3)	0.0202 (5)	
H2A	0.6604	0.2748	0.9447	0.024*	
H2B	0.6891	0.3377	0.8472	0.024*	
C3	0.7407 (2)	0.38245 (14)	1.0797 (3)	0.0168 (5)	
C4	0.8329 (2)	0.34500 (16)	1.2344 (4)	0.0217 (5)	
H4	0.8462	0.2875	1.2364	0.026*	
C5	0.9058 (2)	0.38981 (17)	1.3858 (3)	0.0221 (5)	
H5	0.9700	0.3635	1.4897	0.026*	
C6	0.8845 (2)	0.47317 (16)	1.3845 (3)	0.0209 (5)	
H6	0.9324	0.5040	1.4883	0.025*	
C7	0.7926 (2)	0.51166 (15)	1.2307 (3)	0.0167 (5)	
H7	0.7780	0.5687	1.2309	0.020*	
C8	0.7217 (2)	0.46789 (13)	1.0763 (3)	0.0144 (4)	
C9	0.6244 (2)	0.50717 (14)	0.9095 (3)	0.0130 (4)	
C10	0.6259 (2)	0.59116 (14)	0.8755 (3)	0.0131 (4)	
C11	0.5282 (2)	0.62902 (15)	0.7197 (3)	0.0133 (4)	
C12	0.4277 (2)	0.57892 (14)	0.5984 (3)	0.0142 (4)	
C13	0.4282 (2)	0.49398 (15)	0.6262 (3)	0.0162 (5)	
C14	0.5264 (2)	0.45766 (14)	0.7787 (3)	0.0166 (4)	
C15	0.7262 (2)	0.64517 (14)	0.9918 (3)	0.0137 (4)	
C16	0.3254 (2)	0.61565 (15)	0.4420 (3)	0.0174 (5)	
C17	0.3244 (2)	0.44469 (14)	0.4820 (3)	0.0143 (4)	
C18	0.3423 (2)	0.39889 (15)	0.3792 (3)	0.0183 (5)	
H18	0.4216	0.3967	0.4075	0.022*	
C19	0.2471 (2)	0.35638 (14)	0.2367 (3)	0.0185 (5)	
H19	0.2601	0.3253	0.1670	0.022*	
C20	0.1321 (2)	0.36031 (14)	0.1981 (3)	0.0166 (5)	
C21	0.1114 (2)	0.40498 (17)	0.2974 (3)	0.0238 (5)	
H21	0.0318	0.4071	0.2682	0.029*	
C22	0.2085 (2)	0.44712 (17)	0.4412 (3)	0.0212 (5)	
H22	0.1953	0.4776	0.5114	0.025*	

### Atomic displacement parameters (Å^2^)

**Table:**

	*U*^11^	*U*^22^	*U*^33^	*U*^12^	*U*^13^	*U*^23^
Br1	0.02085 (13)	0.02220 (13)	0.01646 (13)	−0.00648 (12)	0.00229 (10)	−0.00716 (12)
N1	0.0175 (11)	0.0161 (11)	0.0163 (11)	0.0000 (8)	0.0068 (10)	0.0001 (8)
N2	0.0141 (10)	0.0118 (9)	0.0155 (11)	0.0002 (8)	0.0036 (9)	0.0002 (8)
N3	0.0210 (11)	0.0174 (10)	0.0201 (11)	0.0007 (9)	0.0048 (10)	0.0001 (9)
C1	0.0180 (12)	0.0132 (11)	0.0159 (11)	−0.0025 (9)	0.0079 (10)	−0.0014 (9)
C2	0.0217 (12)	0.0143 (11)	0.0213 (13)	−0.0016 (10)	0.0114 (11)	−0.0011 (10)
C3	0.0180 (12)	0.0132 (11)	0.0187 (12)	−0.0004 (9)	0.0110 (11)	0.0018 (9)
C4	0.0171 (11)	0.0202 (12)	0.0250 (13)	0.0042 (10)	0.0115 (11)	0.0090 (11)
C5	0.0191 (12)	0.0238 (13)	0.0197 (12)	0.0009 (10)	0.0100 (11)	0.0105 (10)
C6	0.0184 (11)	0.0261 (13)	0.0142 (11)	−0.0025 (10)	0.0079 (10)	0.0019 (10)
C7	0.0159 (10)	0.0162 (11)	0.0165 (11)	−0.0016 (9)	0.0091 (10)	0.0009 (9)
C8	0.0127 (10)	0.0134 (11)	0.0160 (11)	−0.0005 (9)	0.0081 (9)	0.0024 (9)
C9	0.0121 (11)	0.0131 (10)	0.0138 (11)	0.0014 (9)	0.0079 (10)	−0.0001 (9)
C10	0.0126 (10)	0.0133 (10)	0.0129 (10)	−0.0014 (9)	0.0075 (9)	−0.0030 (9)
C11	0.0130 (11)	0.0132 (10)	0.0140 (11)	−0.0003 (9)	0.0084 (10)	−0.0019 (9)
C12	0.0136 (10)	0.0140 (11)	0.0114 (11)	0.0009 (8)	0.0057 (9)	0.0006 (8)
C13	0.0146 (11)	0.0131 (11)	0.0157 (11)	−0.0013 (9)	0.0066 (10)	−0.0029 (10)
C14	0.0173 (10)	0.0139 (11)	0.0165 (11)	−0.0023 (9)	0.0093 (10)	−0.0007 (9)
C15	0.0140 (11)	0.0116 (10)	0.0123 (11)	0.0025 (9)	0.0063 (10)	0.0007 (9)
C16	0.0173 (11)	0.0133 (10)	0.0176 (11)	−0.0026 (9)	0.0085 (10)	−0.0045 (10)
C17	0.0149 (10)	0.0104 (10)	0.0121 (10)	−0.0007 (9)	0.0055 (9)	0.0000 (9)
C18	0.0143 (11)	0.0174 (11)	0.0193 (12)	−0.0003 (9)	0.0082 (10)	−0.0023 (10)
C19	0.0208 (11)	0.0168 (12)	0.0167 (11)	−0.0005 (10)	0.0108 (10)	−0.0031 (10)
C20	0.0165 (11)	0.0115 (10)	0.0115 (11)	−0.0049 (9)	0.0032 (9)	−0.0006 (9)
C21	0.0161 (11)	0.0328 (14)	0.0193 (12)	−0.0061 (11)	0.0091 (10)	−0.0056 (11)
C22	0.0187 (12)	0.0270 (13)	0.0180 (12)	−0.0043 (10)	0.0111 (10)	−0.0071 (10)

### Geometric parameters (Å, °)

**Table:**

Br1—C20	1.898 (2)	C7—H7	0.9500
N1—C15	1.149 (3)	C8—C9	1.485 (3)
N2—C11	1.359 (3)	C9—C10	1.408 (3)
N2—H1	0.93 (3)	C9—C14	1.415 (3)
N2—H2	0.88 (4)	C10—C11	1.420 (3)
N3—C16	1.152 (4)	C10—C15	1.435 (3)
C1—C14	1.511 (3)	C11—C12	1.410 (3)
C1—C2	1.528 (4)	C12—C13	1.407 (3)
C1—H1A	0.9900	C12—C16	1.434 (3)
C1—H1B	0.9900	C13—C14	1.395 (3)
C2—C3	1.503 (3)	C13—C17	1.489 (3)
C2—H2A	0.9900	C17—C22	1.389 (3)
C2—H2B	0.9900	C17—C18	1.390 (3)
C3—C4	1.390 (4)	C18—C19	1.384 (3)
C3—C8	1.410 (3)	C18—H18	0.9500
C4—C5	1.387 (4)	C19—C20	1.387 (3)
C4—H4	0.9500	C19—H19	0.9500
C5—C6	1.385 (4)	C20—C21	1.376 (4)
C5—H5	0.9500	C21—C22	1.395 (4)
C6—C7	1.392 (4)	C21—H21	0.9500
C6—H6	0.9500	C22—H22	0.9500
C7—C8	1.395 (3)		
			
C11—N2—H1	119 (2)	C9—C10—C11	122.2 (2)
C11—N2—H2	117 (2)	C9—C10—C15	123.5 (2)
H1—N2—H2	115 (3)	C11—C10—C15	114.3 (2)
C14—C1—C2	110.4 (2)	N2—C11—C12	120.5 (2)
C14—C1—H1A	109.6	N2—C11—C10	122.3 (2)
C2—C1—H1A	109.6	C12—C11—C10	117.1 (2)
C14—C1—H1B	109.6	C11—C12—C13	121.2 (2)
C2—C1—H1B	109.6	C11—C12—C16	118.7 (2)
H1A—C1—H1B	108.1	C13—C12—C16	120.1 (2)
C3—C2—C1	109.2 (2)	C14—C13—C12	120.6 (2)
C3—C2—H2A	109.8	C14—C13—C17	122.1 (2)
C1—C2—H2A	109.8	C12—C13—C17	117.1 (2)
C3—C2—H2B	109.8	C13—C14—C9	119.7 (2)
C1—C2—H2B	109.8	C13—C14—C1	121.9 (2)
H2A—C2—H2B	108.3	C9—C14—C1	118.2 (2)
C4—C3—C8	119.3 (2)	N1—C15—C10	173.1 (2)
C4—C3—C2	121.4 (2)	N3—C16—C12	177.3 (3)
C8—C3—C2	119.3 (2)	C22—C17—C18	119.1 (2)
C3—C4—C5	121.4 (2)	C22—C17—C13	121.9 (2)
C3—C4—H4	119.3	C18—C17—C13	118.9 (2)
C5—C4—H4	119.3	C19—C18—C17	121.4 (2)
C6—C5—C4	119.5 (2)	C19—C18—H18	119.3
C6—C5—H5	120.2	C17—C18—H18	119.3
C4—C5—H5	120.2	C18—C19—C20	118.3 (2)
C5—C6—C7	119.8 (2)	C18—C19—H19	120.9
C5—C6—H6	120.1	C20—C19—H19	120.9
C7—C6—H6	120.1	C21—C20—C19	121.7 (2)
C6—C7—C8	121.2 (2)	C21—C20—Br1	119.50 (19)
C6—C7—H7	119.4	C19—C20—Br1	118.77 (18)
C8—C7—H7	119.4	C20—C21—C22	119.3 (2)
C7—C8—C3	118.6 (2)	C20—C21—H21	120.4
C7—C8—C9	122.7 (2)	C22—C21—H21	120.4
C3—C8—C9	118.7 (2)	C17—C22—C21	120.2 (2)
C10—C9—C14	118.7 (2)	C17—C22—H22	119.9
C10—C9—C8	122.9 (2)	C21—C22—H22	119.9
C14—C9—C8	118.3 (2)		
			
C14—C1—C2—C3	56.3 (3)	C10—C11—C12—C16	177.9 (2)
C1—C2—C3—C4	141.2 (2)	C11—C12—C13—C14	2.9 (3)
C1—C2—C3—C8	−37.6 (3)	C16—C12—C13—C14	−179.2 (2)
C8—C3—C4—C5	0.6 (4)	C11—C12—C13—C17	−173.1 (2)
C2—C3—C4—C5	−178.1 (2)	C16—C12—C13—C17	4.9 (3)
C3—C4—C5—C6	1.8 (4)	C12—C13—C14—C9	2.4 (3)
C4—C5—C6—C7	−1.9 (4)	C17—C13—C14—C9	178.1 (2)
C5—C6—C7—C8	−0.6 (4)	C12—C13—C14—C1	178.8 (2)
C6—C7—C8—C3	3.0 (3)	C17—C13—C14—C1	−5.5 (4)
C6—C7—C8—C9	−178.9 (2)	C10—C9—C14—C13	−6.0 (3)
C4—C3—C8—C7	−3.0 (3)	C8—C9—C14—C13	174.4 (2)
C2—C3—C8—C7	175.8 (2)	C10—C9—C14—C1	177.4 (2)
C4—C3—C8—C9	178.9 (2)	C8—C9—C14—C1	−2.2 (3)
C2—C3—C8—C9	−2.3 (3)	C2—C1—C14—C13	145.4 (2)
C7—C8—C9—C10	26.4 (3)	C2—C1—C14—C9	−38.2 (3)
C3—C8—C9—C10	−155.6 (2)	C14—C13—C17—C22	108.9 (3)
C7—C8—C9—C14	−154.1 (2)	C12—C13—C17—C22	−75.2 (3)
C3—C8—C9—C14	24.0 (3)	C14—C13—C17—C18	−75.0 (3)
C14—C9—C10—C11	4.7 (3)	C12—C13—C17—C18	100.9 (3)
C8—C9—C10—C11	−175.7 (2)	C22—C17—C18—C19	0.6 (4)
C14—C9—C10—C15	−173.7 (2)	C13—C17—C18—C19	−175.6 (2)
C8—C9—C10—C15	5.8 (3)	C17—C18—C19—C20	−0.1 (4)
C9—C10—C11—N2	−176.9 (2)	C18—C19—C20—C21	0.0 (4)
C15—C10—C11—N2	1.7 (3)	C18—C19—C20—Br1	179.03 (17)
C9—C10—C11—C12	0.3 (3)	C19—C20—C21—C22	−0.4 (4)
C15—C10—C11—C12	178.9 (2)	Br1—C20—C21—C22	−179.4 (2)
N2—C11—C12—C13	173.1 (2)	C18—C17—C22—C21	−0.9 (4)
C10—C11—C12—C13	−4.2 (3)	C13—C17—C22—C21	175.1 (2)
N2—C11—C12—C16	−4.8 (3)	C20—C21—C22—C17	0.8 (4)

### Hydrogen-bond geometry (Å, °)

**Table:**

*D*—H···*A*	*D*—H	H···*A*	*D*···*A*	*D*—H···*A*
N2—H1···N1^i^	0.93 (3)	2.23 (3)	3.097 (3)	155 (3)
N2—H2···N3^ii^	0.88 (4)	2.54 (4)	3.307 (3)	147 (3)

Symmetry codes: (i) *x*−1/2, −*y*+3/2, *z*−1/2; (ii) *x*+1/2, −*y*+3/2, *z*+1/2.
